# Durvalumab Prolongs Overall Survival, Whereas Radiation Dose Escalation > 66 Gy Might Improve Long-Term Local Control in Unresectable NSCLC Stage III: Updated Analysis of the Austrian Radio-Oncological Lung Cancer Study Association Registry (ALLSTAR)

**DOI:** 10.3390/cancers17091443

**Published:** 2025-04-25

**Authors:** Franz Zehentmayr, Petra Feurstein, Elvis Ruznic, Brigitte Langer, Brane Grambozov, Marisa Klebermass, Alexandra Hochreiter, Ayurzana Purevdorj, Georg Gruber, Danijela Minasch, Barbara Breitfelder, Claudia Steffal, Karoline Kirchhammer, Heidi Stranzl, Falk Röder, Karin Dieckmann

**Affiliations:** 1 Department of Radiation Oncology, Paracelsus Medical University, 5020 Salzburg, Austria; e.ruznic@salk.at (E.R.); b.grambozov@salk.at (B.G.); a.hochreiter@salk.at (A.H.); f.roeder@salk.at (F.R.); 2 Department of Radiation Oncology, Klinikum Ottakring, 1160 Vienna, Austria; doktor@petra-feurstein.at (P.F.); dr.brigitte.langer@aon.at (B.L.); marisa.klebermass@gesundheitsverbund.at (M.K.); 3 Department of Radiation Oncology, Klinikum Hietzing-Rosenhügel, 1130 Vienna, Austria; ayurzana.purevdorj@gesundheitsverbund.at; 4 Department of Radiation Oncology, Ordensklinikum Linz, 4020 Linz, Austria; georg.gruber@ordensklinikum.at; 5 Department of Radiation Oncology, Comprehensive Cancer Centre, Medical University Innsbruck, 6020 Innsbruck, Austria; danijela.minasch@tirol-kliniken.at; 6 Department of Radiation Oncology, Klinikum Krems, 3500 Krems, Austria; barbarab@utanet.at; 7 Department of Radiation Oncology, Klinikum Favoriten, 1100 Vienna, Austria; claudia.steffal@gesundheitsverbund.at; 8 Department of Radiation Oncology, Klinikum Klagenfurt, 9020 Klagenfurt, Austria; karoline.kirchhammer@kabeg.at; 9 Department of Radiation Oncology, Comprehensive Cancer Centre, Medizinische Universität Graz, 8036 Graz, Austria; heidi.stranzl@medunigraz.at; 10 Department of Radiation Oncology, Comprehensive Cancer Centre, Medical University Vienna, 1090 Vienna, Austria; karin.dieckmann@akhwien.at

**Keywords:** non-operable NSCLC, durvalumab, local control, total radiation dose, overall survival

## Abstract

Introduction: Long-term analysis of PACIFIC revealed the clinical benefit of chemoradiotherapy combined with Durvalumab for unresectable non-small-cell lung cancer (NSCLC) stage III. ALLSTAR is a prospective registry aimed at validating the PACIFIC regimen in daily practice in Austria. Patients and Methods: Patients were eligible if they had pathologically confirmed unresectable NSCLC III with a curative treatment option. The endpoints for this analysis were overall survival (OS), updated local control (LC), and progression-free survival (PFS). Results: The 2- and 3-year LC rates in patients who received total radiation doses > 66 Gy were 80% and 75%, respectively, which were superior to the standard treatment (65% and 54%; *p*-value 0.085). This benefit was even more pronounced in Durvalumab patients with 2- and 3-year LC rates of 82% and 79% with >66 Gy (*p*-value 0.068). The 2- and 3-year OS with Durvalumab was 71% and 63%, respectively, compared to 58% and 44% without Durvalumab (HR 0.552; 95%-CI 0.347–0.881; *p*-value 0.011). Patients who were treated with Durvalumab also had a significantly longer 2- and 3-year PFS (56% and 48%) than those without (35% and 20%; HR 0.469; 95%-CI 0.312–0.707; *p*-value < 0.001). Pulmonary side effects were observed in 66/188 (35%) patients, with one case of grades 4 and 5 each. Oesophageal toxicity grade 1–3 occurred in 93/188 (49%) cases. Conclusion: The updated ALLSTAR analysis demonstrated sustained benefit of Durvalumab for OS and PFS, as well as a possible long-term benefit of radiation dose escalation > 66 Gy on LC.

## Simple Summary

Lung cancer is still a leading cause of cancer death worldwide. According to randomized trials, the combination of chemotherapy, radiation, and immunotherapy has led to a substantial improvement in long-term clinical outcomes. The Austrian Radio-Oncological Lung Cancer Study Association Registry (ALLSTAR) is a prospective nationwide registry aimed at validating this combination therapy, which has come to be termed as the so-called *PACIFIC regimen*. The endpoints for this analysis were overall survival (OS), updates for local control (LC), and progression-free survival (PFS). The results from this real-world data analysis demonstrated that tumour relapse in the lungs was less likely with high radiation doses and that immunotherapy—in this case Durvalumab—prolongs disease-free and overall survival. Therefore, the PACIFIC regimen shows its benefits in routine practice outside clinical trials.

## 1. Introduction

Lung cancer accounts for 12% of all cancer diagnosis and 18% of cancer related deaths worldwide [1]. The proportion of non-small-cell lung cancer (NSCLC) amounts to approximately 80% of lung cancers with one-third presenting already in locally advanced stages, i.e., UICC IIIa to IIIc [1].

In the pre-immunotherapy era, 5-year overall survival with chemoradiotherapy (CRT) was in the range of 15–30% for UICC stage III patients [2], and the median overall survival (mOS) was less than 2.5 years [3]. In this respect, the publication of the PACIFIC data constitutes a breakthrough in the field of locally advanced NSCLC III (LA-NSCLC) with 5-year OS rates of 43% for patients receiving Durvalumab [4,5,6]. In this prospective phase III trial, 713 patients were randomized in a 2:1 ratio. Patients who received Durvalumab had a median PFS of 17.2 months compared to 5.6 months without. OS at 5 years was 43% for all patients receiving Durvalumab—and even 50% for PD-L1-positive patients—compared to 33% in the placebo arm. Toxicity rates did not differ significantly between the interventional and the placebo arm [4,5,6]. Long-term outcome data also demonstrate enduring disease control and prolonged OS with this treatment regimen [4].

LA-NSCLC consists of several sub-entities that entail a variety of treatment approaches [7]. As only 2% of the patients qualify for randomized control trials (RCTs) [8], real-world data (RWD) that demonstrate the efficacy of a prospectively tested treatment regimen in daily oncological routine are increasingly required by funding bodies. The rising number of RWD sets published in the wake of the PACIFIC trial consistently corroborate the sustained clinical benefits achieved with immunotherapy [9,10,11,12,13,14,15,16,17,18,19]. In contrast, one Japanese RWD study [20] found no difference between chemoradioimmunotherapy (CRIT) and CRT with respect to progression-free survival (PFS) and OS. However, the results presented by this group reveal a highly significant benefit of Durvalumab treatment with respect to local control (LC). This somewhat astonishing finding contradicts not only a previous publication by our group [21] but also a post hoc analysis of the PACIFIC data demonstrating LC rates similar to historical series [22,23,24,25,26]. Hence, ALLSTAR belongs to the small number of studies reporting on this endpoint [10,18,20,22,27], which may exert crucial influence on long-term OS [28]. In this context, dose escalation comes into play. Although RTOG 0617 demonstrated that conventionally fractionated CRT with total doses of 74 Gy was disadvantageous for survival [3], a meta-analysis including about 2000 patients showed that moderate dose escalation may be beneficial in terms of OS [29]. In the context of the PACIFIC trial, the radiation schemes with total doses between 54 and 66 Gy were not described extensively so the effect of this regimen on LC is in need of further clarification.

While in a previous publication we reported improved LC with dose escalation > 66 Gy and prolonged PFS with Durvalumab [21], the current analysis presents OS together with updated LC and PFS. Data extraction for the current analysis was performed approximately four years after the first patient was recruited and one year after data cutoff for the abovementioned prior publication.

## 2. Methods

### 2.1. Study Design

ALLSTAR is a registry that was designed with the idea of collecting real-world data on unresectable stage III NSCLC in Austria (approval by the ethics committee of the Federal State of Salzburg, no. 1002/2019). It differs from other RWD studies [11,14,15] in two major aspects: firstly, the approach in the current registry was prospective with clearcut inclusion criteria, and, secondly, the patients were not part of an early-access programme (EAP). Eligibility criteria, as reported previously [21], included patients with pathologically confirmed inoperable NSCLC UICC stage III (according to TNM version 8). Based on a consensually taken decision by the thoracic tumour board with all related disciplines involved, patients had to have a curative treatment option. Details on diagnostic work-up and follow-up are published elsewhere [21]. The primary objective of ALLSTAR was to assess how far currently used chemoradiation schedules combined with immunotherapy impact clinical outcome and toxicity.

### 2.2. Radiochemoimmunotherapy

In analogy to PACIFIC [5,6], patients received CRT followed by immunotherapy if they were PD-L1-positive. However, as reported previously [21], some patients were treated with immunotherapy at the discretion of the local tumour board regardless of their PD-L1 status. Although concomitant chemoradiotherapy (cCRT) is regarded as the standard of care [2,23,24,25,26], most patients (129/188; 69%) in ALLSTAR received sequential chemoradiotherapy (sCRT). Details on treatment modalities were reported in a prior publication [21]. In brief, the technical pre-requisites for administering thoracic radiotherapy had to be 3D RT at least. Total radiation doses of 60–66 Gy in 2 Gy fractions were considered standard of care (SoC). In this regard, patients who received >66 Gy were defined as a dose escalation group. As radiation treatment schedules differed between institutions, the biologically equivalent dose in 2 Gy fractions (EQD2) was calculated in order to compare the variety of treatment approaches [30,31] with D for total dose, d for dose per fraction, and α/β assumed as 10 for tumour tissue:

(1) $$EQD2=D\frac{d+\frac{\alpha }{\beta }}{2+\frac{\alpha }{\beta }}$$

Although prospective RCTs [2,23,24,25,26] revealed an outcome advantage for patients who received CRT in the concomitant compared to sequential mode, the latter approach was also allowed in the current registry. As for immunotherapy, patients with programmed death ligand 1 (PD-L1) > 1% were considered positive and therefore eligible for Durvalumab treatment according to EMA [32].

### 2.3. Endpoints and Statistics

The basis for the current investigation was the data extraction performed on the 17th of May 2024, which is 50 months after recruiting the first patient. All time-to-event estimations were performed with the Kaplan–Meier method using pathological diagnosis as index date. The clinical endpoints of the present analysis are overall survival, updated LC as defined by Machtay [28], and PFS. Additionally, we report updated toxicity rates (CTCAEv5). Adverse events of special interest (AESI) were pulmonary and oesophageal side effects. These two terms comprise a variety of organ-specific side effects, which cannot always be clearly differentiated in daily clinical practice [21]. While subgroup comparisons were performed by log-rank testing, the Mann–Whitney U test was used to compare toxicity data between immunotherapy and non-immunotherapy groups. In line with the updated results from PACIFIC-R [16], the following potential prognosticators were tested for their influence on the abovementioned endpoints: UICC stage (IIIa versus IIIb/c), non-squamous-cell carcinoma (SCC) versus squamous cell carcinoma (SCC), PD-L1 (yes or no), chemoradiotherapy sequence (sequential or concomitant), 42-day limit for the start of Durvalumab therapy.

## 3. Results

### 3.1. Patients

Between March 2020 and April 2023, 12/14 (86%) Austrian radiation oncology centres recruited 188 patients who had at least one follow-up visit and were therefore eligible for the current analysis. Except for gross tumour volume (GTV) and the substantially higher total radiation dose in the immunotherapy group, baseline patient characteristics were—as reported previously [21]—well balanced between the immunotherapy and non-immunotherapy groups (Supplementary Table S1). By 17 May 2024, which was the cutoff for the current analysis, 98/188 (52%) patients were still alive (77/130 in the immunotherapy group, 21/58 in the non-immunotherapy group), 71/188 (38%) patients had died (44/130 in the immunotherapy group, 27/58 in the non-immunotherapy group), and 19/188 (10%) were lost to follow-up (9/130 in the immunotherapy group, 10/58 in the non-immunotherapy group).

When stratifying the whole patient cohort by total radiation dose with 66 Gy as cutoff, it becomes obvious that the groups differ in the distribution of gender, Durvalumab treatment, and size of GTV_Tumour_ (Table 1).

### 3.2. Radiochemoimmunotherapy

The total median EQD2 to the tumour was 66 Gy (range: 32.5–100) in the immunotherapy group compared to 60 Gy (range: 24.8–100) in the patients without immunotherapy. While in one patient (0.5%) the total radiation dose was 24.8 Gy EQD2, eight (4%) received 32.5 Gy. The median PTV of 717 mL in these patients was higher by a factor of almost 2.5 compared to the rest of the cohort (median PTV of 317 mL). All of them had compromised lung function with median FEV1 of 60% with one patient on long-term oxygen treatment. The therapeutic strategy of the multidisciplinary tumour board was to give induction chemotherapy in order to cause tumour shrinkage so that the patient could be offered a curative approach. On the other end of the radiation dose spectrum, nine patients (5%) from one single institution received 100 Gy EQD2 using a simultaneous integrated boost concept. The boost PTV was defined—at the discretion of the treating radiation oncologist—as the area at “highest risk” within the tumour, whereas the surrounding tumour received 60 Gy EQD2. As for chemotherapy, platinum-doublets were combined with pemetrexed, taxane, gemcitabine, or vinorelbine according to histology (Supplementary Table S2). After CRT, 130/188 patients received immunotherapy, the majority of which (113/130 patients) were treated with Durvalumab [21] (Supplementary Table S3). PD-L1 tests were available in 173/188 (92%) patients (Supplementary Table S1). The median interval between CRT and Durvalumab was 14 days (range: 1–65 days). While 98/113 (87%) patients started Durvalumab within 42 days, in 5/113 (4%) patients the interval was up to 65 days. For 10/113 (9%) patients, no data were available. The median number of Durvalumab cycles was 13 (range: 1–38), and 34 of 113 (30%) patients had 21 to 38 cycles and were therefore regarded as having finished treatment. Cortisone treatment served as proxy for toxicity-related therapy discontinuation. This analysis revealed that 30/113 (27%) received cortisone for one of the following reasons: pneumonitis (20), COPD exacerbation (3), lung fibrosis (2), joint pain (1), pancreatitis (2), hypophysitis (1), colitis (1). Discontinuation because of progression occurred in 32/113 (28%) patients. The median empirical time interval from initiation of Durvalumab until cancer relapse was 11.1 months. The median time interval from initiation of Durvalumab until cancer relapse was 11.1 months. Thereafter, 21/32 (66%) patients received the following types of anticancer treatment: chemotherapy (5), immunotherapy other than Durvalumab (7), chemotherapy combined with immunotherapy other than Durvalumab (2), radiotherapy alone (1), radiochemotherapy (2), radiochemotherapy combined with immunotherapy other than Durvalumab (1), tyrosine kinase inhibitors (3). In 11/32 (34%) patients, no data on further treatment were available.

### 3.3. Local Control, Overall Survival, and Progression-Free Survival

The median follow-up was 25.1 months (range: 3.3–52.1) in the whole cohort and 30.3 months (range: 11.0–52.1) in patients alive at data extraction.

#### 3.3.1. Local Control

**Figure 1:**
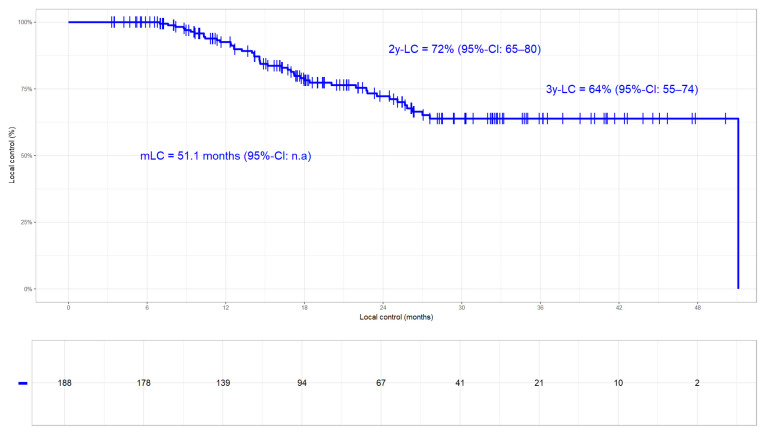
Local control (LC). The median LC was 51.1 months with 72% and 64% at 2 years and 3 years, respectively.

**Figure 2:**
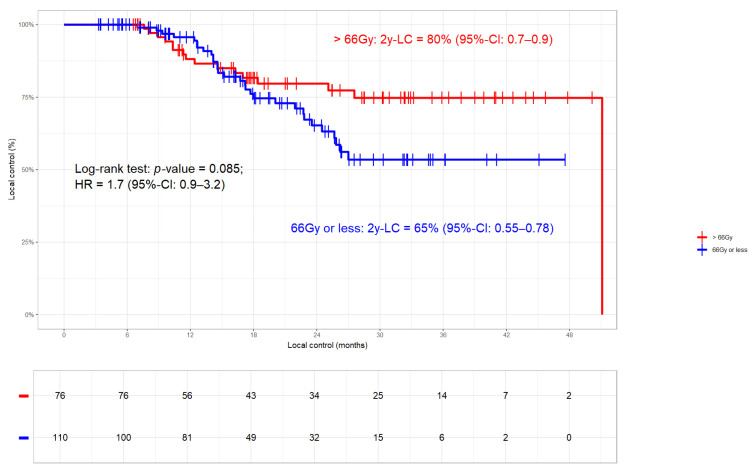
Local control (LC) in dependence of total radiation dose (>/≤ 66 Gy; N = 186). Patients who received >66 Gy had better LC than those without (log-rank *p*-value 0.085; HR 1.7; 95%-CI 0.9–3.2). In two patients, detailed information on total radiation dose was not available.

**Figure 3:**
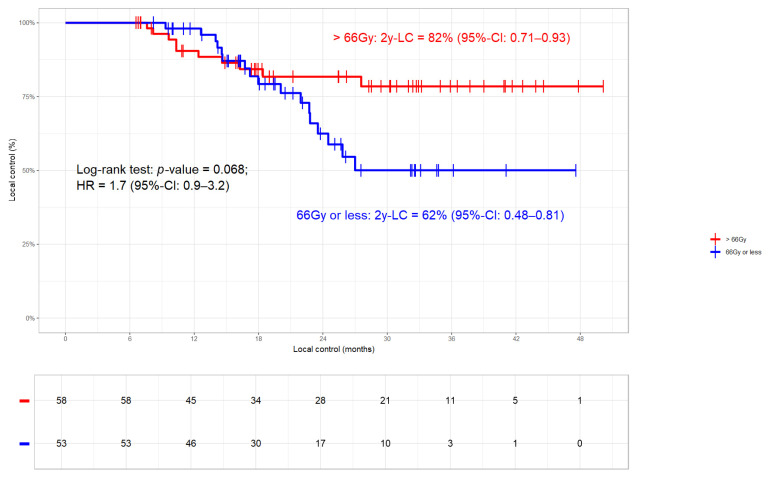
Local control (LC) in dependence of total radiation dose (>/≤66 Gy) in patients who received Durvalumab (N = 111). Patients who received >66 Gy had better LC than those without (log-rank *p*-value 0.068). In two patients, detailed information on total radiation dose was missing.

In the whole cohort, 46/188 (24%) local progressions occurred. The LC rates were 72% and 64% at 2 years and 3 years, respectively (median LC 51.1 months, Figure 1). While 16 (9%) patients had an isolated relapse in the primary tumour, five (3%) failed exclusively in the lymph nodes and 25 (13%) relapsed in both sites (Table 2). Patients who received >66 Gy had better LC (2- and 3-year LC rates of 80% and 75%, respectively) compared to those with SoC radiation dose (2- and 3-year LC rates of 65% and 54%, respectively; *p*-value 0.085; Figure 2). In two patients, detailed information on total radiation dose was missing. The fact that moderate radiation dose escalation led to improved LC compared to SoC could be corroborated in the 113 Durvalumab patients, 26 (23%) of which had progressed (2- and 3-year LC rates of 82% and 79% versus 63% and 50%, respectively; *p*-value 0.068; Figure 3). Nine patients had a relapse in the primary tumor only, two had isolated lymph node failures and fifteen relapsed in both sites (Table 1). In order to detect potentially confounding variables that might have an influence on LC, we used the same stratification parameters as PACIFIC-R [16], i.e., UICC disease stage, histology, sequence of chemoradiation, PD-L1 status, and the 42-day limit for starting Durvalumab, in a time-to-event analysis. While non-SCC histology (median not reached) proved to be beneficial compared to SCC (median of 27.6 months; *p*-value < 0.001; Supplementary Figure S1), none of the other parameters had a significant impact on LC. Regarding the above mentioned differences between the two radiation dose groups (Table 2), MVA revealed that the only significant factor that impacts LC is histology (N = 188; HR 2.234 95%-CI 1.399–3.566; *p*-value < 0.001; Table 3) with total radiation dose (>/<66 Gy) showing borderline significance (*p*-value = 0.059). This was corroborated when testing the same variables in the high-dose and SoC subgroups (Supplementary Tables S5 and S6) with histology being the only significant parameter (Supplementary Figures S4 and S5).

#### 3.3.2. Overall Survival

**Figure 4:**
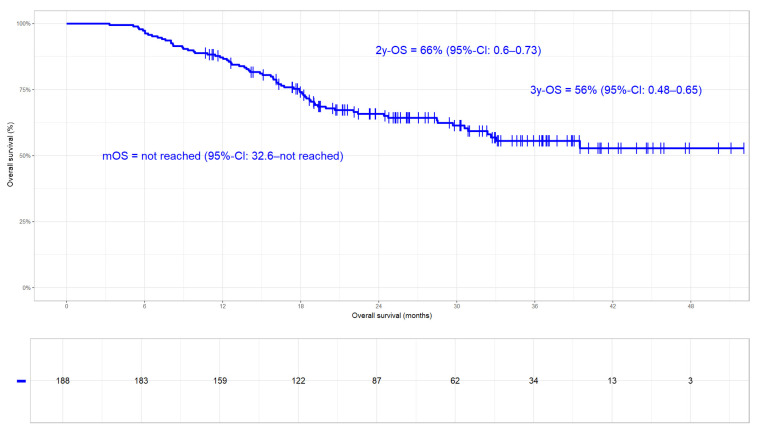
Overall survival (OS) in the whole cohort (N = 188, median not reached). OS rates at 2 and 3 years were 66% and 56%, respectively.

**Figure 5:**
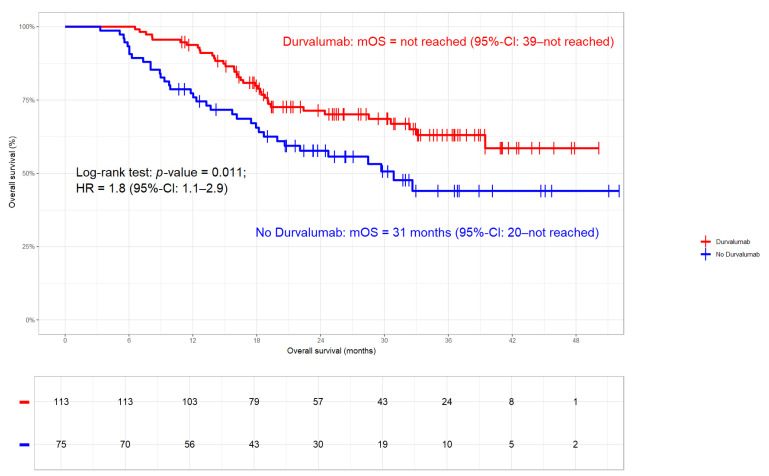
Overall survival (OS) stratified by Durvalumab. In patients treated with Durvalumab, median OS was not reached, while in those without immunotherapy, it was 30.9 months (HR 1.8; 95%-CI 1.1–2.9; *p*-value 0.011).

In the whole cohort, 71/188 (38%) patients had died at the time of data extraction. With survival rates of 66% and 56% at 2 years and 3 years, respectively, the median was not reached (Figure 4). Patients who received Durvalumab had significantly better OS compared to those without. While in the first group the median was not reached (2- and 3-year rates of 71% and 63%, respectively), the latter had an mOS of 30.9 months (95%-CI: 20.0–not reached; HR 1.8; 95%-CI 1.1–2.9; *p*-value 0.011; Figure 5) and 2- and 3-year rates of 58% and 44%, respectively. Again, we tested UICC disease stage, histology, sequence of chemoradiation, PD-L1 status, and the 42-day limit in a time-to-event analysis for their potential influence on OS [16]. None of these parameters had a significant impact on OS.

#### 3.3.3. Progression Free Survival

**Figure 6:**
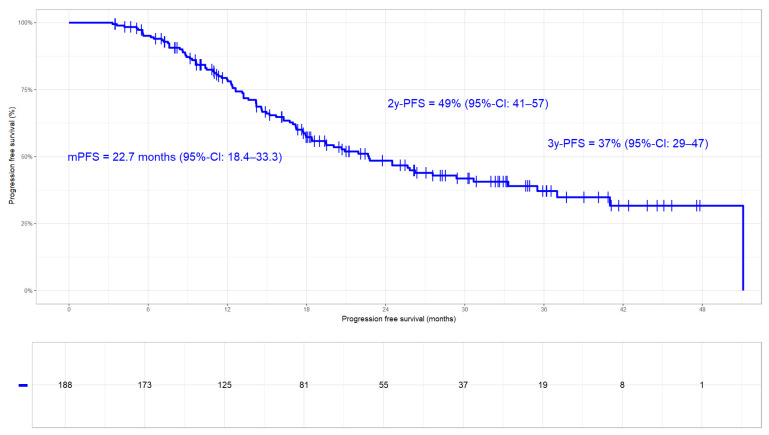
Progression-free survival in the whole cohort: median PFS 22.7 months (95%-CI 18.4–33.3 months). PFS rates at 2 and 3 years were 49% and 37%, respectively.

**Figure 7:**
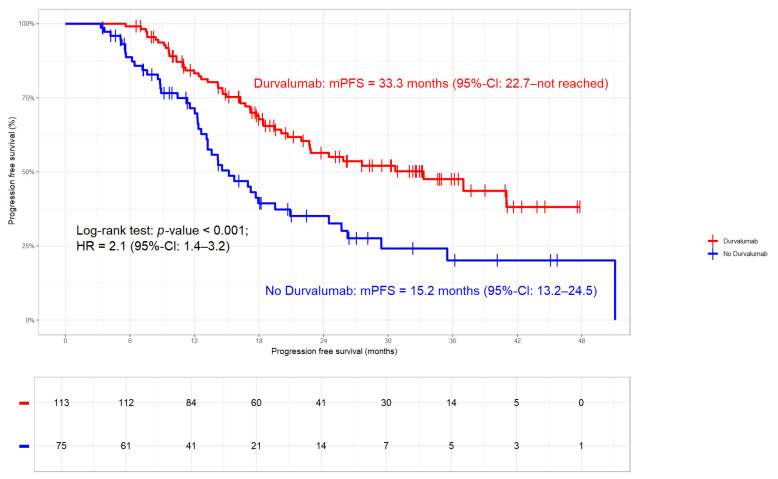
Progression-free survival stratified by Durvalumab. In patients treated with Durvalumab, median PFS was 33.3 months (95%-CI: 22.7—not reached) compared to 15.2 months (95%-CI: 13.2–24.5) in those without (HR 2.1; 95%-CI 1.4–3.2; *p*-value < 0.001).

With 94 (50%) progressions in the whole cohort of 188 patients, the overall updated median PFS was 22.7 months (95%-CI: 18.4–33.3 months; Figure 6) and the 2- and 3-year rates were 49% and 37%, respectively. In line with a prior publication [21], patients who were treated with Durvalumab had a significantly longer PFS (median: 33.3 months; 95%-CI 20.9–45.7 months; 56% and 48% at 2 years and 3 years, respectively) than those without (median: 15.2 months; 13.2–24.5 months; 35% and 20% at 2 years and 3 years, respectively; HR 2.1; 95%-CI: 1.4–3.2; *p*-value < 0.001; Figure 7). While histology, sequence of chemoradiation, PD-L1 status, and the 42-day limit had no significant impact on PFS, patients with UICC stage IIIa had better mPFS (median not reached) compared to UICC stage IIIb/c patients (median 18.0 months; 95%-CI: 15.7–27.6 months; HR 1.9 95%-CI 1.2–3.1; *p*-value 0.006; Supplementary Figure S2).

### 3.4. Toxicity

**Figure 8:**
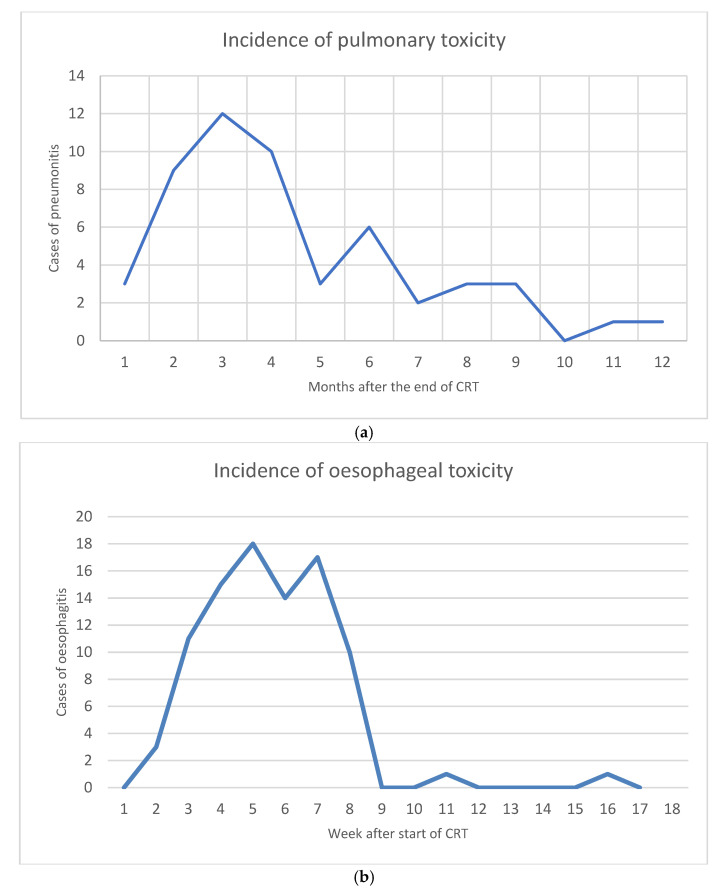
(**a**) Dynamics in the incidence of pulmonary toxicity during the first year after CRT. (**b**) Dynamics in the incidence of oesophageal toxicity during four months after the start of CRT.

The main AESIs were oesophageal and pulmonary toxicity. With 46/130 (35%) and 20/58 (34%) cases, respectively, the occurrence of pulmonary toxicity did not differ significantly between groups (Mann–Whitney test *p*-value 0.741, Table 4). The onset of pulmonary toxicity reached a peak approximately 3 months after the end of CRT (Figure 8a). One case of grade 4 pulmonary toxicity occurred in a patient in the non-immunotherapy group. This patient was treated with Osimertinib for brain metastases after having completed chemoradiation with 60.5 Gy EQD2 six months before. The only patient with grade 5 lung toxicity died 3.2 months after finishing sequential chemoradiation. In this case, systemic treatment consisted of three cycles of induction chemotherapy with Carboplatinum plus Taxane before RT and three cycles of Durvalumab after thoracic irradiation. The GTVTumour had a size of 7 mL and was irradiated to a total EQD2 of 100 Gy. The dose to the 45 mL GTVInvolved lymph nodes was 49 Gy EQD2. Elective nodal irradiation of 32.5 Gy was administered to a GTV of 429 mL. Two weeks after the third cycle of Durvalumab, the patient presented with dyspnea and radiographic signs of pneumonitis. Steroid treatment was initiated but another three weeks later the patients was admitted to hospital again with bacterial superinfection. She was treated with mycophenolate mofetil but refused invasive respiratory measures. Since a therapy-related cause of death could not be entirely excluded, this patient was registered as grade 5 pulmonary toxicity. In the context of high-dose radiation, the correlation between GTV size together with Durvalumab and pulmonary toxicity is of special concern. In the 75 patients who received total radiation doses beyond 66 Gy, 24 (32%) developed grade 1 to 3 pneumonitis. Additionally, the abovementioned case of lethal pneumonitis was also treated with high-dose radiation. This updated analysis corroborates the initial finding in as far as a correlation between GTV_Tumour_ and pulmonary toxicity could not be found (Supplementary Figure S3). As for oesophageal toxicity, 63/130 (48%) cases grades 1–3 occurred in the immunotherapy group, while 30/58 (52%) cases were observed in patients without immunotherapy (Mann–Whitney U test *p*-value 0.774, Table 2). The majority of these cases (95%) occurred within the first 7 weeks after start of (chemo)radiation (Figure 8b). Apart from the abovementioned AESIs, a list of other side effects is added in Supplementary Table S4, most of which were grade 1 or 2. This list also includes eleven cases of grade 3 toxicities: COPD exacerbation (3), joint pain (1), dermatitis (1), nausea (1), hemoptysis (2), hypophysitis (1), and pancreatitis (2).

## 4. Discussion

In line with a previous report [21], this updated analysis of the ALLSTAR registry also demonstrated improved LC with high-dose radiation >66 Gy. Furthermore, Durvalumab prolonged mOS and mPFS, which is consistent with the results of the PACIFIC trial [4,5,6].

With a median follow-up of 25.1 months for all patients and 30.3 months for those alive at data extraction, ALLSTAR is on the upper edge of the literature ranging from 14 to 39 months [16,17,18,20,33,34] (Supplementary Table S7). ALLSTAR is one of the few studies [10,18,20,22,27] that report LC as a separate endpoint. With 2- and 3-year rates of 80% and 75%, respectively, LC was nominally superior in the patient group that received a total dose of 66 Gy EQD2 or more, which is quite similar to the results from another dose escalation study [35]. Based on 1-year outcome data in small study cohorts, some authors put forward the notion that Durvalumab helps to improve LC [18,20,27]. This viewpoint contradicts a post hoc analysis of PACIFIC [22], which stated that LC rates achieved by Durvalumab do not differ substantially from historical RCTs [3,23,24,25,26]. In this regard, optimal total radiation dose and fractionation are still a matter of debate [11]. In the pre-immunotherapy era, RTOG 0617 showed a detrimental effect of 74 Gy EQD23, while a meta-analysis favoured moderate dose-escalation by accelerated regimens [29]. While 40% of the patients in ALLSTAR underwent dose escalation >66 Gy, the proportion in other RWD studies is 10% or lower [11,12,33,36], which precludes further comparisons in this respect. Nevertheless, the updated results from ALLSTAR together with previous reports [37,38] suggest that moderate radiation dose escalation may be beneficial in times of immune-checkpoint inhibitor (ICI) treatment. Of note, an interesting study on SBRT for patients with residual disease after conventional thoracic irradiation revealed excellent local control rates [39,40]. This approach could be an alternative treatment strategy for patients not eligible for immunotherapy. In accordance with the results from the PACIFIC trial, patients who received Durvalumab had significantly better OS than those without [4,6]. Of note, with 30.9 months, the mOS in the non-immunotherapy group was in the same range as the 28.7 months in the RTOG 0617 trial [3]. The 2- and 3-year OS rates in ALLSTAR (71% and 63%) and the number of deaths (38%) were comparable to the updated results of other RWD studies [9,10,14,16,18,27,34,36]. In contrast, a Japanese study reports a non-significant 12-month OS difference between patients with and without Durvalumab [20]. As for confounding factors that may exert an influence on OS, the 5-year report of the PACIFIC trial [4] and PACIFIC-KR [14] showed that ECOG impacts OS, which is corroborated by our results. SCC, however, did not have a negative influence on OS in our analysis, whereas in PACIFIC [4] and PACIFIC-R [16] it did. In line with the PACIFIC trial, the current update showed that patients who received Durvalumab had a significantly prolonged mPFS of 33.3 months, which appears substantially longer than published data [4,9,10,12,14,16]. One has to bear in mind, however, that the index date in ALLSTAR is the day of pathological diagnosis and not initiation of Durvalumab, so time-to-event analysis yield results are seemingly 4–5 months longer. Hence, the upper edge of the mPFS range between 20.1 and 26 months in other RWDs [9,10,11,12,14] almost falls in the same range as ours. Nevertheless, together with 56% and 48% at 2 and 3 years, respectively, the PFS for Durvalumab patients in ALLSTAR appears comparatively favourable. This finding could be potentially explained by the short interval between the end of CRT and the start of ICI. Of note, in the abovementioned single-centre Japanese study conflicting results were reported, showing no difference in 1-year PFS between the CRT and CRIT groups [20]. As for prognosticators, similar to our results, the 5-year update of PACIFIC and some RWD studies showed that UICC stage [4,14,16,17] and histology [4] had a significant impact on mPFS.

Clinically relevant pulmonary toxicity grade 2 or higher occurred in 22% of the cases with a peak incidence 12 weeks after the end of CRT. Both the incidence rate known from published data at a range between 8.6% and 38.5% [9,11,14,15,17,18] and the latency [18,27] are similar to other studies. In this context, we agree with the authors of PACIFIC-R11 and other groups [9,14,15] that the dividing line between immunotherapy- or radiation-induced pneumonitis is hard to draw. Of note, one study reports grade 2 or higher pulmonary side effects at a substantially higher rate of 65.7%, possibly owing to the extremely comprehensive definition [41]. As for severe pulmonary toxicity, the 2.7% grade 3–4 in ALLSTAR was in the scope of other RWDs [11,17] but less than half the rate of the original PACIFIC trial [5,6]. Finally, the very small fraction of 0.5% lethal lung damage is also found in comparable studies [14,18,19]. With regard to oesophageal toxicity, the current report complements previous data [21]. Grade 3 or higher oesophagitis occurred at a rate and latency comparable to the literature [18]. With 2% thyroiditis and 1% hypophysitis (Supplementary Table S4), the rates of endocrinopathies were markedly lower in ALLSTAR than in literature reports [42], which could well be the result of a measurement bias as the focus in the present study was placed on the main side effects of thoracic chemoradioimmunotherapy, i.e., pulmonary and oesophageal toxicities.

A meta-analysis lists seven major differences between RWD and prospective RCTs [8], four of which can also be found in our cohort: median age > 65 years, patients with ECOG > 1 are included, use of sCRT, and the median cycle number of Durvalumab is <20. Hence, we agree with Wang et al. in as far as these differences in patient population do not allow RCT results to be simply transferred to daily clinical routine but underline the necessity for RWD sets. The median age at lung cancer diagnosis in clinical practice is usually around 70 years [8], which is 6 years higher than in the PACIFIC trial [5,6]. This is also reflected by ALLSTAR with 40% aged 70+ and other RWD studies with a substantial proportion of patients in this age group [17,19,20]. ALLSTAR included nine (6%) ECOG 2–3 patients, which is almost the same rate as in a Korean study [19] and similar to the Spanish EAP analysis [17]. The only prospective phase II study that focused on Durvalumab in frail patients, i.e., PACIFIC-6, included only three patients with advanced performance scores [43]. These small numbers, which were a point of criticism in regard to PACIFIC-6 [44,45], showcase the difficulty of including patients with ECOG >1 even in registries that are aimed at presenting clinical reality more accurately than RCTs. In this context, sCRT is often administered in elderly and frail patients [8]. While the proportion sCRT cases in RWDs is generally low [9,11,17], in ALLSTAR, approximately two-thirds were treated with sCRT. This is probably not so much based on the frailty of the patient population but rather the fact that about 40% of the patients in ALLSTAR received total doses >66 Gy and sCRT is the preferred mode of treatment for radiation dose escalation regimens. On MVA, however, therapy sequence did not have an influence on any clinical endpoint in ALLSTAR, although a meta-analysis [2] from the pre-immunotherapy era and the Spanish EAP [17] showed that cCRT is better than sCRT. Corresponding to the abovementioned criteria that differentiate RWD from RCTs [8], the median number of Durvalumab cycles in ALLSTAR was 13. The reason for this is probably the difference in patient characteristics with higher age, more co-morbidities, and a reduced general condition in RWD patient collectives, so the fraction of Durvalumab finishers in ALLSTAR is—like in other RWDs [20,33,36]—about one-third, which is markedly lower than the 50% in the original PACIFIC trial16.

According to the EMA decision, Durvalumab treatment in Austria was based on the availability of PD-L1 status. Hence, 92% of the patients in ALLSTAR had a known PD-L1 status, which is markedly higher than in prospective studies [4,43] and other RWD analyses [9,10,11,15,17]. Similar to a Japanese RWD study [20], the updated median interval between the end of CRT and the initiation of Durvalumab was 14 days. Of the 188 patients in ALLSTAR, 87% started Durvalumab within 42 days. Compared to other RWD studies with median intervals of approximately 40 days in most studies [11,15,18,27,36] and up to 72 days in the Spanish cohort [17], the lag time in ALLSTAR is very short. Hence, our results contradict the conclusion drawn by the authors of the Spanish EAP study [17] and O’Leary’s comment on PACIFIC-R [46] stating that early Durvalumab administration is hard to achieve in daily clinical practice. Durvalumab discontinuation for treatment related toxicity, which is a negative prognostic factor for OS [42], occurred in 25% of the ALLSTAR patients. Since this is an estimate by proxy, this number—although in the range of the published literature [14,16,17,20,27,36,42]—should be viewed cautiously. Another 28% of the patients discontinued ICI therapy because of progressive disease, which is also comparable to PACIFIC [4] and other reports [11,14,17,36]. With an empirical rate of 11.1 months, the median time to disease progression in ALLSTAR was nominally more than twice as long as the 4.9-month latency in PACIFIC-R [11]. When taking into consideration the differences in index dates, this discrepancy becomes negligible. Two-thirds of the patients with relapse received subsequent anticancer treatment, consisting of either chemotherapy, immunotherapy other than Durvalumab, RT, or a combination of the three modalities. This is slightly higher than the 50% in the PACIFIC trial [4].

### Study Limitations

Although 12 of the 14 Austrian radiation oncology departments contributed to ALLSTAR, the data distribution might be biased as 85% of the patient population was from three institutions, meaning that more active centres with a higher affinity for new therapy concepts are potentially over-represented. Nevertheless, the results from this study are comparable to the largest RWD set [11,16]. A second limitation, due to the lack of resources, concerns the less stringent follow-up compared to RCTs. Because of the COVID-19 pandemic, some follow-up intervals may have been additionally extended. Therefore, LC, OS, and PFS are seemingly higher compared to prospective RCTs and toxicity rates might—especially in lower grades—appear smaller. In general, the data quality in RWD studies is not directly comparable to prospective randomized control trials, which is an inherent shortcoming. This type of analysis, however, may provide valuable insights by demonstrating how results gained in prospective RCTs inform daily clinical practice. Finally, GTV_Tumour_ and EQD2 differed substantially between groups. These two parameters may have an impact on clinical outcome, especially on LC. On multivariate analysis, however, only EQD2 showed borderline significance, while GTV did not have an impact (Table 3).

## 5. Conclusions

This updated analysis of the ALLSTAR registry demonstrated that dose escalation > 66 Gy in NSCLC III may have a long-term benefit for LC. Durvalumab therapy after completion of CRT shows sustained anticancer response with prolonged PFS and OS, whereby the current data contribute to the validation of the PACIFIC regimen as the standard of care treatment for NSCLC III.

## Figures and Tables

**Table 1:** Baseline characteristics stratified by total radiation dose (>/< 66 Gy).

Baseline Characteristics				
		<66 Gy N = 110 (%)	>66 Gy N = 78 (%)	*p*-Value
Gender	male	60 (55)	54 (69)	0.038
	female	50 (45)	24 (30)	
Age (years)	median	67	67	0.222
	range	45–91	36–84	
Smoking status	never	6 (5)	7 (9)	0.883
	ex	65 (59)	43 (55)	
	current	39 (35)	28 (36)	
ECOG	0–1	102 (93)	74 (95)	0.866
	2–3	8 (7)	4 (5)	
UICC	IIIa	33 (30)	34 (44)	0.061
	IIIb	56 (51)	27 (35)	
	IIIc	21 (19)	17 (22)	
Histology	nonSCC	63 (57)	47 (60)	0.793
	SCC	47 (42)	31 (40)	
PDL-1	<1%	28 (25)	15 (19)	0.371
	>1%	73 (66)	57 (73)	
	unknown	9 (8)	6 (8)	
CRT sequence	sCRT	69 (63)	61 (78)	0.032
	cCRT	33 (30)	14 (18)	
	unknown	8 (7)	3 (4)	
Durvalumab	yes	57 (52)	18 (23)	<0.001
	no	53 (48)	60 (77)	
Tumour GTV (mL)	median	78.6	19	<0.001
	range	1–784	1–282	

**Table 2:** Sites of local relapse.

Local Relapses		
	All Patients N = 188 (%)	Durvalumab Patients N = 113 (%)
Tumour (isolated)	16 (9)	9 (8)
Tumour + lymph nodes	25 (13)	15 (13)
Lymph nodes (isolated)	5 (3)	2 (2)

**Table 3:** Multivariate analysis for variables that may impact local control revealed that histology was the only significant parameter with total radiation dose (>/<66 Gy) showing a strong trend.

Baseline Characteristics		
	UVA	MVA
Gender	0.111	n.s.
Age	0.526	n.s.
Smoking status	0.116	n.s.
ECOG	0.400	n.s.
UICC	0.523	n.s.
Histology	0.001	<0.001
PDL-1	0.982	n.s.
CRT sequence	0.213	n.s.
Durvalumab	0.262	n.s.
Tumour GTV	0.152	n.s.
Total radiation dose (>/<66 Gy)	0.059	n.s.

**Table 4:** Toxicity.

Toxicity				
		Immunotherapy N = 130 (%)	No immunotherapy N = 58 (%)	*p*-Value
Oesophagitis	Grade 1	17 (13)	7 (12)	0.774
	Grade 2	43 (33)	22 (38)	
	Grade 3	3 (2)	1 (2)	
	Grade 4	0 (0)	0 (0)	
	Grade 5	0 (0)	0 (0)	
Pneumonitis	Grade 1	18 (14)	5 (9)	0.741
	Grade 2	26 (20)	12 (21)	
	Grade 3	1 (1)	2 (2)	
	Grade 4	0 (0)	1 (2)	
	Grade 5	1 (1)	0 (0)	
Haematologic	any grade	2 (2)	2 (3)	n.a.
Other	any grade	30 (23)	6 (10)	n.a.

## Data Availability

The original contributions presented in this study are included in the article. Further inquiries can be directed to the corresponding author.

## Supplementary Materials

The following supporting information can be downloaded at: https://www.mdpi.com/article/10.3390/cancers17091443/s1. Supplementary Figures S1 to S5; Supplementary Tables S1 to S7.

## Abbreviations

AESI	adverse event of special interest
ALLSTAR	Austrian Radio-Oncological Lung Cancer Study Association Registry
CRIT	chemoradioimmunotherapy
CRT	chemoradiotherapy
cCRT	concomitant chemoradiotherapy
EAP	early-access programme
EQD2	biologically equivalent dose in 2 Gy fractions
GTV	gross tumour volume
ICI	immune-checkpoint inhibitor
IMRT	intensity-modulated radiotherapy
LA-NSCLC	locally advanced non-small-cell lung cancer
LC	local control
MVA	multivariate analysis
OS	overall survival
PD-L1	programmed death ligand 1
PFS	progression-free survival
RCT	randomized control trial
RT	radiotherapy
RWD	real-world data
SCC	squamous cell carcinoma
SoC	standard of care
sCRT	sequential chemoradiotherapy
VMAT	volumetric arc therapy
