# Supplementary material for: Durvalumab Prolongs Overall Survival, Whereas Radiation Dose Escalation > 66 Gy Might Improve Long-Term Local Control in Unresectable NSCLC Stage III: Updated Analysis of the Austrian Radio-Oncological Lung Cancer Study Association Registry (ALLSTAR)
Source: Cancers (Basel). 2025 Apr 25;17(9):1443. doi: 10.3390/cancers17091443 (PMC12070846; doi:10.3390/cancers17091443)
Supplement: Supplementary file 1 [file cancers-17-01443-s001.zip › cancers-3489743-supplementary.pdf]

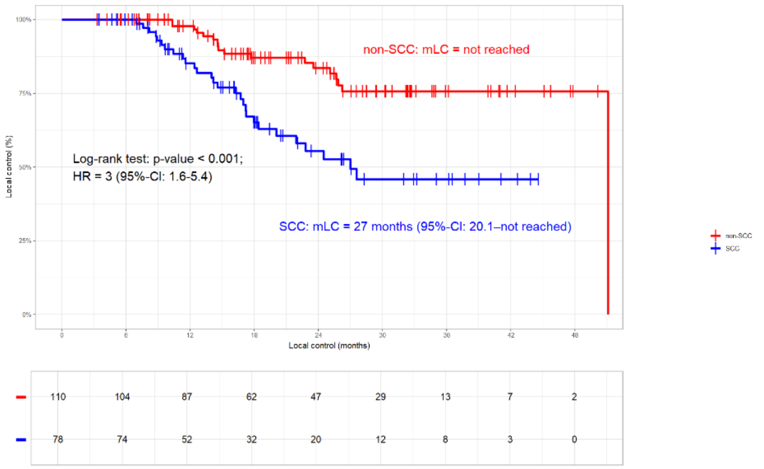

Supplementary Figure S1. While the median LC for patients with non-SCC histology was not reached, it was 27.6 months (95%-CI: 20.1 – not reached) for SCC patients (p-value < 0.001; HR 3.0; 95%-CI 1.6 – 5.4).

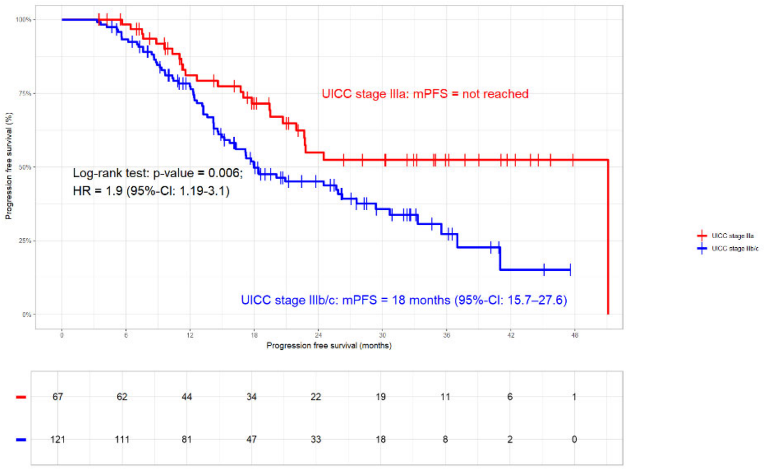

Supplementary Figure S2. While the median PFS for patients with UICC stage IIIa was not reached, it was 18.0 months (95%-CI: 15.7 – 27.6 for UICC stage IIIb or IIIc patients (p-value 0.006; HR 1.9; 95%-CI 1.2 – 3.1).

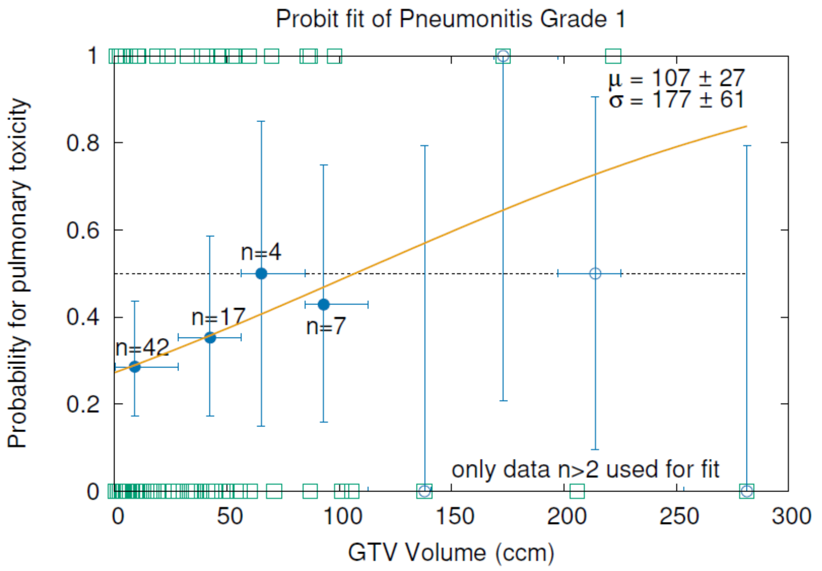

Supplementary Figure S3. This plot shows the correlation between GTVTumour and the probability for pulmonary toxicity. Of the 75 patients, five had a GTV of 138 to 281.5 mL, whereas the other 70 patients had GTV sizes up to 105 mL. These latter were divided in four group intervals of the same size. Although the fitted curve seems to show an increased risk for pulmonary toxicity with higher GTV, this correlation was not significant, which corroborates previous findings.

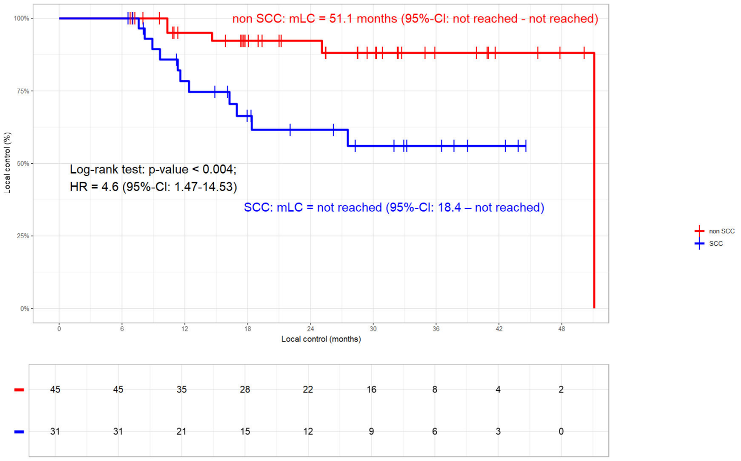

Supplementary Figure S4. In the high dose group (>66 Gy; n = 110) non-squamous cell histology (non-SCC) positively impacted LC.

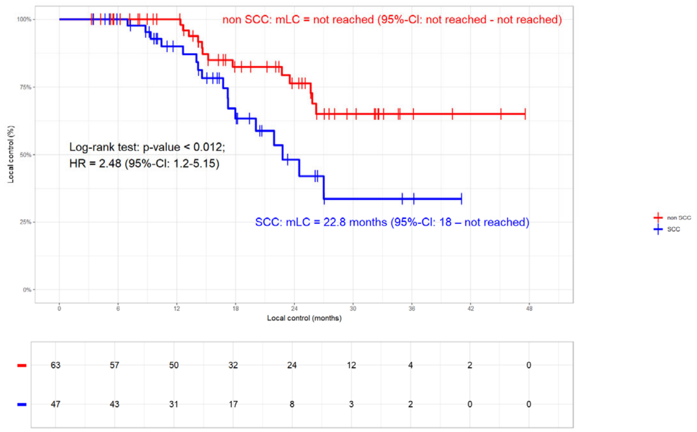

Supplementary Figure S5. In the SoC group (<66 Gy; n = 110) non-squamous cell histology (non-SCC) positively impacted LC.

Supplementary Table S1. Patient characteristics (AC = adenocarcinoma, SCC = squamous cell carcinoma, NOS = not otherwise specified)

Patients N = 188				
		**Immunotherapy n = 130 (%)**	**No immunotherapy n = 58 (%)**	**p-Value**
Sex	male	78 (60)	36 (62)	0.788
	female	52 (40)	22 (38)	
Age (years)	median	67	68	0.955
	range	41 - 84	36 - 91	
Smoking status	never	10 (8)	3 (5)	0.556
	ex	75 (58)	33 (57)	
	current	45 (35)	22 (38)	
ECOG	0-1	123 (95)	53 (91)	0.403
	2-3	7 (5)	5 (9)	
Histology	AC	72 (55)	26 (45)	0.089
	SCC	52 (40)	26 (45)	
	NOS	5 (4)	6 (10)	
PDL-1	< 1%	17 (13)	26 (45)	<0.001
	> 1%	105 (81)	25 (43)	
	unknown	8 (6)	7 (12)	
T-stage	Tis	1 (1)	1 (2)	0.179
	1	21 (16)	5 (9)	
	2	15 (12)	8 (14)	
	3	41 (32)	15 (26)	
	4	50 (38)	29 (50)	
	unknown	2 (2)	0 (0)	
N-stage	0	9 (7)	6 (10)	0.428
	1	7 (5)	9 (16)	
	2	86 (66)	28 (48)	
	3	28 (22)	15 (26)	
M-stage	0	130 (100)	58 (100)	n.a.
	1	0 (0)	0 (0)	
UICC	IIIa	45 (35)	22 (38)	0.795
	IIIb	59 (45)	24 (41)	
	IIIc	26 (20)	12 (21)	

Supplementary Table S2. Chemotherapy combinations used prior or concomitantly to radiotherapy (RT).

Chemotherapy N = 129				
**Chemotherapy prior to RT**		**Immunotherapy n = 92 (%)**	**No Immunotherapy n = 37 (%)**	**p-Value**
Substances	carboplatinum/pemetrexed	38 (41)	12 (32)	0.541
	carboplatinum/taxane	10* (11)	5 (14)	
	carboplatinum/gemcitabine	26 (28)	8 (22)	
	carboplatinum/vinorelbine	9 (10)	4* (11)	
	cisplatinum/pemetrexed	6* (7)	5 (14)	
	cisplatinum/taxane	1 (1)	1 (3)	
	cisplatinum/gemcitabine	0 (0)	2 (5)	
	cisplatinum/vinorelbine	2 (2)	0 (0)	
Cycles	median	2	3	0.539
	range	1-4	1-6	
**Chemotherapy simultaneous to RT**		**Immunotherapy n = 35 (%)**	**No Immunotherapy n = 12 (%)**	**p-Value**
Substances	carboplatinum/pemetrexed	7 (20)	2 (17)	0.345
	carboplatinum/taxane	2 (6)	0 (0)	
	carboplatinum/gemcitabine	0 (0)	0 (0)	
	carboplatinum/vinorelbine	2 (6)	4 (33)	
	cisplatinum/pemetrexed	13 (37)	2 (17)	
	cisplatinum/taxane	0 (0)	0 (0)	
	cisplatinum/gemcitabine	0 (0)	0 (0)	
	cisplatinum/vinorelbine	11 (31)	4 (33)	
Cycles	median	3	2	0.303
	range	1-4	2-4	

*One patient in this group also received chemotherapy after radiotherapy.

Supplementary Table S3. Treatment characteristics (CRT = chemoradiotherapy, VMAT = volumetric arc therapy, IMRT = intensity modulated radiotherapy, EQD2 = biologically equivalent dose in 2 Gy fractions, GTV = gross tumour volume).

Treatment N = 188					
			**Immunotherapy n = 130 (%)**	**No immunotherapy n = 58* (%)**	**p-Value**
Treatment sequence	concomitant CRT		35 (27)	12 (21)	0.501
	sequential CRT		92 (71)**	37 (64)***	
	radio(immuno)therapy		3 (3)	9 (16)	
Immune Checkpoint inhibitors	Durvalumab		113 (87)	x	n.a.
	Pembrolizumab		13 (10)	x	
	Nivolumab		1 (1)	x	
	Atezolizumab		3 (2)	x	
RT Technique	VMAT/IMRT		125 (96)	57 (98)	0.446
	3-D		5 (4)	1 (2)	
Tumor	EQD2	median	66	60	<0.001
		range	32.5 - 100	24.8 - 100	
	GTV	median	44.7	79.2	0.004
		range	0.24 - 483,8	1 – 784.1	
Lymph nodes	EQD2	median	57.3	50	0.179
		range	31.3 - 70	24 – 81.3	
	GTV	median	33,75	53	0.209
		range	1 - 408,1	1,9 - 473	
Elective nodal irradiation	EQD2	median	32.5	51.6	0.242
		range	32.5 - 60	32.5 - 54	
	GTV	median	86	137	0.374
		range	2 - 429	83.5 - 265	

* This group includes one patient who received osimertinib.
** Two patients in this group received chemotherapy after radiotherapy.
*** One patient in this group received chemotherapy after radiotherapy.

Supplementary Table S4. List of other toxicities.

List of other toxicities		
	**Immunotherapy**	**No immunotherapy**
	**n = 130**	**n = 58**
Anorexia	1 (1)	0 (0)
Arteriitis temporalis	1 (1)	0 (0)
Colitis	1 (1)	0 (0)
COPD exacerbation	5 (4)	0 (0)
Cough	2 (2)	0 (0)
Dermatitis	1 (1)	0 (0)
Fatigue	1 (1)	1 (2)
Hemoptysis	1 (1)	1 (2)
Hepatitis	1 (1)	0 (0)
Hypophysitis	1 (1)	0 (0)
Lung fibrosis	1 (1)	0 (0)
Nausea	1 (1)	0 (0)
Odynophagia	0 (0)	1 (2)
Pain	3 (2)	2 (3)
Pancreatitis	4 (3)	0 (0)
Pericarditis	1 (1)	0 (0)
Polyneuropathy	1 (1)	0 (0)
Rash	1 (1)	1 (2)
Thyroiditis	3 (2)	0 (0)

Supplementary Table S5. In the high dose subgroup MVA revealed histology as the only parameter that had an influence on LC.

>66 Gy		
	**UVA**	**MVA**
Gender	0.246	n.s
Age	0.670	n.s
Smoking status	0.378	n.s
ECOG	0.413	n.s
UICC	0.247	n.s
Histology	<0,001	<0.001
PDL-1	0.127	n.s.
CRT sequence	0.018	n.s.
Durvalumab	0.381	n.s.
Tumour GTV	0.442	n.s.

Supplementary Table S6. In the SoC subgroup MVA revealed histology as the only parameter that had an influence on LC.

<66 Gy		
	**UVA**	**MVA**
Age	0.063	n.s.
Gender	0.540	n.s.
ECOG	0.694	n.s.
Smoking status	0.261	n.s.
UICC	0.562	n.s.
Histology	0.013	0.016
PD-L1	0.197	n.s.
CRT sequence	0.743	n.s.
Durvalumab	0.848	n.s.
Tumour GTV	0.592	n.s.

Supplementary Table S7. RWD studies in patients with NSCLC stage III treated with chemoradioimmunotherapy.

Author [Ref.]	N	Median age	cCRT or sCRT	Total radiation dose (Gy)^	Interval (days)^	Duration of ICI (median)	Median FUP (months)	LC	OS	PFS	Lung toxicity	Oesophagus toxicity
Abe [20]	66	70	cCRT	54-66	no ICI	no ICI	26	1y: 62%	1y: 89%	1y: 57%	°2-5: 34%	n.a.
	44	73	cCRT	60	13	9 cycles	17	1y: 86%	1y: 84%	1y: 58%	°2-5: 37%	n.a.
Borghetti [9]	33	68	cCRT or sCRT	60	no ICI	no ICI	11	n.a.	1y: 56%	1y: 42%	°3-4: 1%	°1-2: 46%
	42	69	cCRT or sCRT	60	47	n.a.	21	n.a.	1y: 83%	1y: 67%		
Bruni [33]	91	64	cCRT	44-66	56	13 cycles	14	79%	1y: 88%	1y: 66%	°3-4: 17%	°1-2: 50%
	64	69	sCRT		51							
Faehling [10]	126	62	sCRT	65	n.a.	n.a.	25	75%	1y: 79%	1y: 56%	°3-5: 10%	n.a.
Filippi [16]	1154	65	cCRT or sCRT	<60 Gy: 43%; >60Gy: 57%	65%: >42	22*	39	n.a.	2y: 72%; 3y: 63%	2y: 50%; 3y: 42%	>°1*	n.a.
Friedes [35]	229	68	cCRT	67	35	32 weeks	39	2y: 81%	median: 35 months	median: 18 months	n.a.	n.a.
Gomez Rueda [17]	244	67	cCRT or sCRT	66	72	44 weeks	22	74%	n.a.	2y: 45%	°2-4: 9%	n.a.
Huang [18]	45	66	cCRT	60-66	no ICI	no ICI	22	1y: 84%	median: 22	median: 9	°3-4: 4%	°3-4: 7%
	39	64	cCRT	60-66	38	13 cycles	15	1y: 55%	median not reached	median: 18	°3-4: 4%	°3-4: 0%
Jung [12]	40	67	cCRT	n.a.	no ICI	no ICI	n.a.	n.a.	n.a.	median: 10	°2-5: 20%	n.a.
	21	66	cCRT		n.a.	n.a.	n.a.	n.a.	n.a.	median not reached	°2-5: 43%	n.a.
Kishi [13]	42	71	cCRT	54-72	no ICI	no ICI	23	57%	2y: 61%	2y: 25%	n.a.	n.a.
	136	70	cCRT	54-72	n.a.	15 cycles	25	71%	2y: 70%	2y: 40%	n.a.	n.a.
Offin [27]	62	66	cCRT	60	45	18 cycles	12	1y: 86%	1y: 85%	1y: 52%	°2-3: 18%	n.a.
Park [14]	157	65	cCRT or sCRT	60	32	19 cycles	19	75%	2y: 71%; 3y: 69%	2y: 52%; 3y: 44%	°2-5:27%	n.a.
Hong [19]	286	68	cCRT	65	n.a.	n.a.	12	94%	median: 37 months	median: 19 months	°2-5: 35%#	n.a.
Preti [15]	118	66	cCRT	n.a.	41	n.a.	18	n.a.	n.a.	n.a.	°2-5: 39%	n.a.
Waterhouse [36]	528	70	cCRT	54-66	47	28 weeks	12	n.a.	2y: 64%	n.a.	°1-5: 13%	n.a.
Current study	130	67	sCRT	66	14	13	25	3y: 64%	3y: 63%	3y: 48%	°2-5: 22%	°2-3: 35%
	58	68		60	no ICI	no ICI			3y: 44%	3y: 20%	°2-4: 25%	°2-3: 40%

References

9. Borghetti, P.; Volpi, G.; Facheris, G.; Cossali, G.; Mataj, E.; La Mattina, S.; Singh, N.; Imbrescia, J.; Bonù, M.L.; Tomasini, D.; et al. Unresectable stage III non-small cell lung cancer: Could durvalumab be safe and effective in real-life clinical scenarios? Results of a single-center experience. *Front. Oncol.* **2023**, *13*, 1208204. https://doi.org/10.3389/fonc.2023.1208204.

10. Faehling, M.; Schumann, C.; Christopoulos, P.; Hoffknecht, P.; Alt, J.; Horn, M.; Eisenmann, S.; Schlenska-Lange, A.; Schütt, P.; Steger, F.; et al. Durvalumab after definitive chemoradiotherapy in locally advanced unresectable non-small cell lung cancer (NSCLC): Real-world data on survival and safety from the German expanded-access program (EAP). *Lung Cancer* **2020**, *150*, 114–122. https://doi.org/10.1016/j.lungcan.2020.10.006.

12. Jung, H.A.; Noh, J.M.; Sun, J.-M.; Lee, S.-H.; Ahn, J.S.; Ahn, M.-J.; Pyo, H.; Ahn, Y.C.; Park, K. Real world data of durvalumab consolidation after chemoradiotherapy in stage III non-small-cell lung cancer. *Lung Cancer* **2020**, *146*, 23–29. https://doi.org/10.1016/j.lungcan.2020.05.035.

13. Kishi, N.; Matsuo, Y.; Shintani, T.; Ogura, M.; Mitsuyoshi, T.; Araki, N.; Fujii, K.; Okumura, S.; Nakamatsu, K.; Kishi, T.; et al. Recurrence patterns and progression-free survival after chemoradiotherapy with or without consolidation durvalumab for stage III non-small cell lung cancer. *J. Radiat. Res.* **2022**, *64*, 142–153. https://doi.org/10.1093/jrr/rrac057.

14. Park, C.-K.; Oh, H.-J.; Kim, Y.-C.; Kim, Y.-H.; Ahn, S.-J.; Jeong, W.G.; Lee, J.Y.; Lee, J.C.; Choi, C.M.; Ji, W.; et al. Korean Real-World Data on Patients with Unresectable Stage III NSCLC Treated with Durvalumab After Chemoradiotherapy: PACIFIC-KR. *J. Thorac. Oncol.* **2023**, *18*, 1042–1054. https://doi.org/10.1016/j.jtho.2023.04.008.

15. Preti, B.T.B.; Sanatani, M.S.; Breadner, D.; Lakkunarajah, S.; Scott, C.; Esmonde-White, C.; McArthur, E.; Rodrigues, G.; Chaudhary, M.; Mutsaers, A.; et al. Real-World Analysis of Durvalumab after Chemoradiation in Stage III Non-Small-Cell Lung Cancer. *Curr. Oncol.* **2023**, *30*, 7713–7721. https://doi.org/10.3390/curroncol30080559.

16. Filippi, A.; Bar, J.; Chouaid, C.; Christoph, D.; Field, J.; Fietkau, R.; Garassino, M.; Garrido, P.; Haakensen, V.; Kao, S.; et al. Real-world outcomes with durvalumab after chemoradiotherapy in patients with unresectable stage III NSCLC: Interim analysis of overall survival from PACIFIC-R. *ESMO Open* **2024**, *9*, 103464. https://doi.org/10.1016/j.esmoop.2024.103464.

17. Rueda, A.G.; Taus, Á.; Álvarez, R.Á.; Bernabé-Caro, R.; Chara, L.; López-Brea, M.; Vilà, L.; González, M.Á.S.; Aldagalán, A.d.B.D.; Herrera, B.E.; et al. The S-REAL study: Spanish real-world data on unresectable stage III NSCLC patients treated with durvalumab after chemoradiotherapy. *Clin. Transl. Oncol.* **2024**, *26*, 1779–1789. https://doi.org/10.1007/s12094-024-03404-9.

18. Huang, Y.; Zhao, J.J.; Soon, Y.Y.; Wong, A.; Aminkeng, F.; Ang, Y.; Asokumaran, Y.; Low, J.L.; Lee, M.; Choo, J.R.E.; et al. Real-world experience of consolidation durvalumab after concurrent chemoradiotherapy in stage III non-small cell lung cancer. *Thorac. Cancer* **2022**, *13*, 3152–3161. https://doi.org/10.1111/1759-7714.14667.

19. Hong, K.S.; Choi, S.H.; Lee, S.Y.; Shin, K.-C.; Jang, J.G.; Kwon, Y.S.; Park, S.H.; Choi, K.-J.; Jung, C.Y.; Eom, J.S.; et al. Durvalumab Consolidation After Chemoradiotherapy in Elderly Patients with Unresectable Stage III NSCLC: A Real-World Multicenter Study. *Clin. Lung Cancer* **2024**, *25*, 354–364. https://doi.org/10.1016/j.cllc.2024.02.006.

20. Abe, T.; Saito, S.; Iino, M.; Aoshika, T.; Ryuno, Y.; Ohta, T.; Igari, M.; Hirai, R.; Kumazaki, Y.; Miura, Y.; et al. Effect of durvalumab on local control after concurrent chemoradiotherapy for locally advanced non-small cell lung cancer in comparison with chemoradiotherapy alone. *Thorac. Cancer* **2020**, *12*, 245–250. https://doi.org/10.1111/1759-7714.13764.

27. Offin, M.; Shaverdian, N.; Rimner, A.; Lobaugh, S.; Shepherd, A.F.; Simone, C.B.; Gelblum, D.Y.; Wu, A.J.; Lee, N.; Kris, M.G.; et al. Clinical outcomes, local–regional control and the role for metastasis-directed therapies in stage III non-small cell lung cancers treated with chemoradiation and durvalumab. *Radiother. Oncol.* **2020**, *149*, 205–211. https://doi.org/10.1016/j.radonc.2020.04.047.

33. Bruni, A.; Scotti, V.; Borghetti, P.; Vagge, S.; Cozzi, S.; D'angelo, E.; Levra, N.G.; Fozza, A.; Taraborrelli, M.; Piperno, G.; et al. Corrigendum: A Real-World, Multicenter, Observational Retrospective Study of

Durvalumab After Concomitant or Sequential Chemoradiation for Unresectable Stage III Non-Small Cell Lung Cancer. *Front. Oncol.* **2021**, *11*, 802949. https://doi.org/10.3389/fonc.2021.802949.

35. Friedes, C.; Iocolano, M.; Lee, S.H.; Li, B.; Duan, L.; Levin, W.P.; Cengel, K.A.; Sun, L.L.; Aggarwal, C.; Marmarelis, M.E.; et al. Patterns of Failure, Low-Volume Relapse, and Subsequent Ablative Management in Locally Advanced Non-Small Cell Lung Cancer Treated with Definitive Chemoradiation and Consolidation Immune Checkpoint Inhibitors. *Int. J. Radiat. Oncol.* **2023**, *118*, 1435–1444. https://doi.org/10.1016/j.ijrobp.2023.10.005.

36. Waterhouse, D.; Yong, C.; Frankart, A.; Brannman, L.; Mulrooney, T.; Robert, N.; Aguilar, K.M.; Ndukum, J.; Cotarla, I. Durvalumab real-world treatment patterns and outcomes in patients with stage III non-small-cell lung cancer treated in a US community setting. *Futur. Oncol.* **2023**, *19*, 1905–1916. https://doi.org/10.2217/fon-2023-0117.
